# Autologous platelet lysates local injections for treatment of tibia non-union with breakage of the nickelclad: a case report

**DOI:** 10.1186/s40064-016-3683-2

**Published:** 2016-11-25

**Authors:** Hong-jiang Jiang, Xun-xiang Tan, Hai-yang Ju, Jin-ping Su, Wei Yan, Xiu-gang Song, Li-wu Qin, Chang-jun Ju, Ling-shuang Wang, De-bao Zou

**Affiliations:** 1 Department of Bone and Joint Surgery, Wendeng Orthopaedic Hospital of Shandong Province, Wendeng, 264400 Shandong China; 2 Zhejiang Xingyue Biotechnology Co. Ltd, Hangzhou, 311121 China

**Keywords:** Tibia and fibula fracture, Non-union, Autologous platelet lysates, Percutaneous injections

## Abstract

**Background:**

Nonunions of the tibia represent challenging orthopedic problems, which require the surgeon to analyze numerous factors and choose an appropriate treatment. This article presents a case report of tibia and fibula fracture patient who failed the internal fixation surgery and successfully recovered after one course of percutaneous autologous platelet lysates injection.

**Case description:**

The patient received an internal nickelclad breakage at 9 months post-surgery but reluctant to accept a second surgery, then autologous platelet lysates (APL) injection which is a less invasive method was recommended. The injections were carried once a week for three times. Radiologic evaluation was conducted every month until recovery.

**Discussion and evaluation:**

To the best of our knowledge, this is the first reported case of tibia delayed union with breakage of the plate resolved with APL injection. Improved clinical evidence was observed at 4 and 6 months after injection. The patient got good bony union at 8 months post-injection. The patient didn’t feel any discomfort postinjection, no complications such as infection, refracture etc. were observed.

**Conclusions:**

APL percutaneous injection could be a new therapeutic option for the treatment of nonunion or delayed healing fractures.

## Background

Bone non-union was defined by US Food and Drugs Administration (FDA) as radiographically visible fracture line after 9 months since injury and delayed union was clinically defined as a fracture with no signs of healing for three consecutive months (Megas 2005). Nonunion of the tibia represents a challenging orthopedic problem, which requires the surgeon to analyze numerous factors and choose an appropriate treatment. Tibia nonunion treatment requires a clear identification of its diagnosis and cause. Nonunion or delayed union has been one of the most severe complications of a fracture surgery, and the current failure rate in non-union surgeries is approximately 20% (Tzioupis and Giannoudis 2007).

Recent advances in cellular and molecular biology have emphasized the role of specific growth factors and cytokines in bone fracture healing (Simpson et al. 2006). Autologous platelet lysates (APL) is a kind of platelet cleavage product which is prepared by high speed centrifugation, freeze-thawing, and supernatant abstraction of the peripheral blood. The activated platelet could release a series of growth factors and cytokines that act synergistically to facilitate MSC attachment, proliferation and differentiation, while the potential effects of APL on bone regeneration need to be investigated. Besides, the exact amounts of key factors such as PDGF-BB, TGF-β1, EGF, IGF-1 need to be detected and APL preparation quality control should be emphasized (Altaie et al. 2016; Chen et al. 2013; Civinini et al. 2011).

This study describes a case report of a patient with fractures of tibia and fibula that failed the surgical treatment and successfully treated with APL.

## Methods

### Patient

**Fig. 1:**
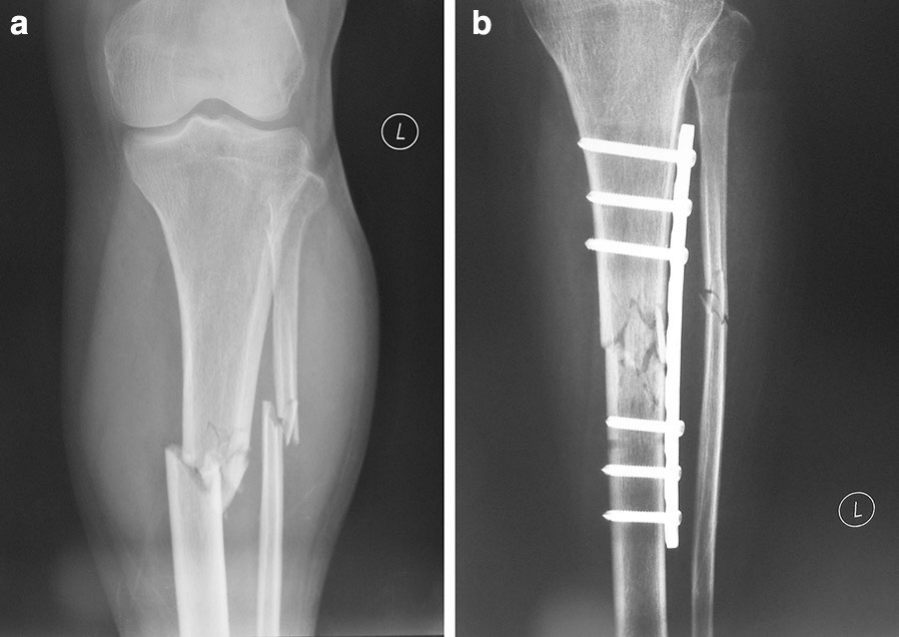
Preoperative radiograph (**a**) and radiograph immediately after incision reduction and internal fixation surgery (**b**) at the first visit to another hospital

A 64-year-old woman was involved in an accident, and she was diagnosed as fractures of left tibia and fibula at Weihai Municipal Hospital at September 17th, 2013. Initial treatment involved incision reduction and a metal plate internal fixation surgery was performed (Fig. 1). The X-ray radiographs were taken every month after the surgery.

**Fig. 2:**
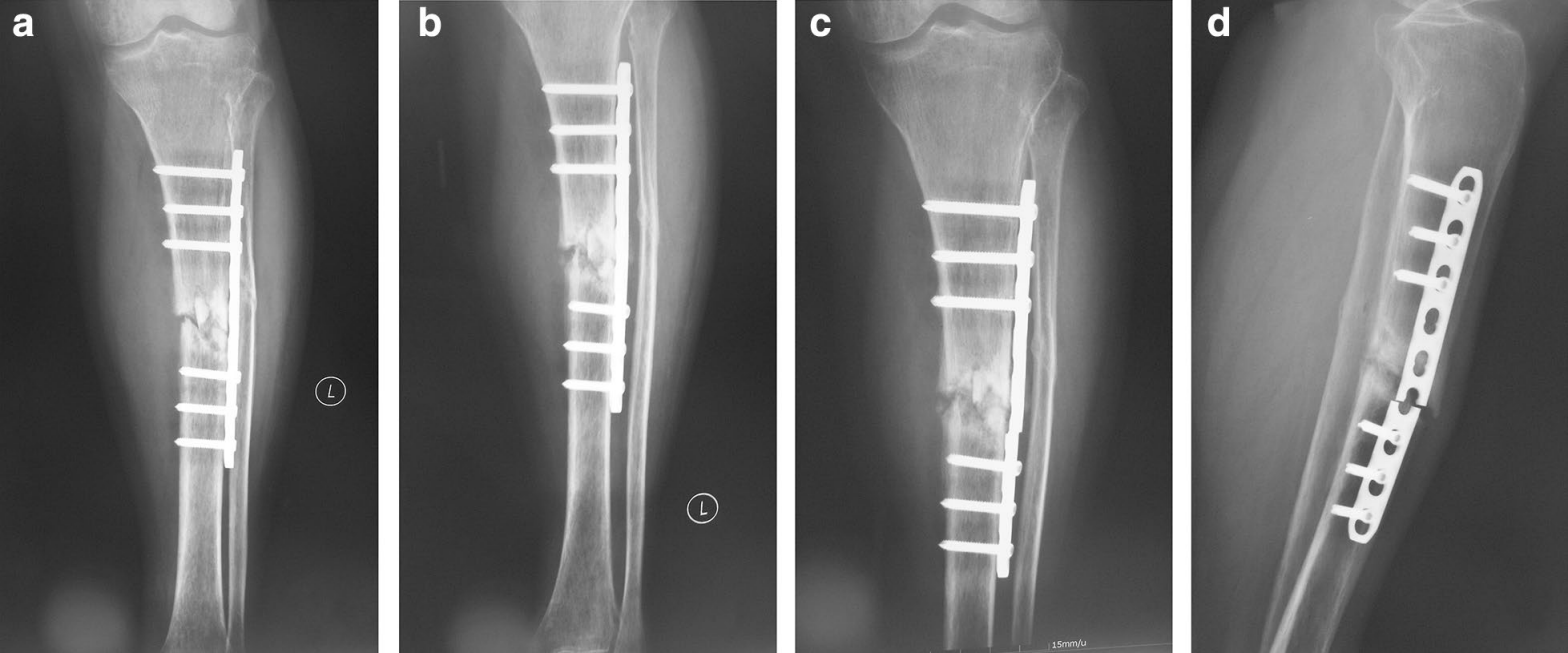
Anteroposterior radiographs of tibia and fibula fracture in 4 months (**a**), 6 months (**b**) and 7 months (**c**) after surgery. Lateral radiograph (**d**) taken 7 months after surgery showing the nickelclad breakage

After 4 months, the radiographs revealed clearly fibula osseous collection and the fibula fracture almost healed, but minimal callus formation was seen and there was a gap in the tibia fracture site (Fig. 2a). The fibula fracture gap disappeared and the fibula got bony union in the sixth month after the surgery. However, through radiologic assessment, no signs of healing in the tibia were observed (Fig. 2b).

On June 7th, 2014 (9 months post injury), the patient went to our station with increasing clinical manifestations of pain at the fracture site over the prior. Radiography revealed tibia fracture stump sclerosis and the breakage of the nickelclad, and tibia non-union was diagnosed (Fig. 2c, d). Incision surgical repair with exchange plate was offered at first because of a visible fracture gap, but the patient preferred less invasive options. Routine blood test didn’t show any abnormality. Since the patient was in a satisfactory nutritional status and her skin and local tissues were in good condition with a straight limb alignment, percutaneous injections of platelet lysates from vein blood concentrate were performed under her permission.

We obtained an informed consent from the patient for participating in clinical therapy regarding percutaneous APL injection in patients with nonunion. The clinical study protocol was approved by the ethics committees of our hospital. After the subject eligibility was confirmed, the patient was registered. Written informed consent for publication of this case report was obtained from the patient.

### Preparation of autologous platelet lysates

The patient was treated by one course of platelet lysates injections, and the platelet lysates were prepared as follows: approximately 40 mL of peripheral vein blood was withdrawn into a syringe containing 1000 IU low molecular weight heparin sodium and then the blood was carefully carried to a local 100 grade purification laboratory for further treatment. After transferred into several centrifuge tubes, the peripheral blood was centrifuged at 200*g* for 20 min at room temperature (Thermo, USA) and then three layers were separated. Platelet rich plasma (PRP) in the middle layer (about 20 mL) was withdrawn and subpackaged into three vacuum tubes (Fisher, USA). Then the tubes were cryopreservation at −80 °C overnight and one of them was resuscitated in the 37 °C water bath kettle in 5 min. After repeatedly freeze thawing more than twice, a variety of growth factors and cytokines were released from the platelet concentrates such as platelet-derived growth factors, transforming growth factor-beta, vascular endothelial growth factors (VEGF) etc.

The thawing and activated plasma was centrifuged at 1700*g* for 6 min (centrifugation radius is 9 cm) to separate the platelet fragmentation in the under layer. The supernatant was filtered to remove cellular debris and WBC contamination is minimized by leukodepletion steps. The leucocyte reduction step is applied by the buffy coat method (Altaie et al. 2016; Singh et al. 2009). 10 mg/mL deoxycycline (APP Pharm, USA) was added into the filtered supernatant with a volume ratio of 1000:1 and then the APL which contains the cocktail of factors released by the platelets was obtained after filtration. The mean volume of APL injected in our series was 5 mL for each infiltration.

### Cytokine detection in peripheral vein blood and APL

The quantitative measurement of PDGF-BB, TGF-β1, IGF-1and EGF concentrations in whole blood and APL were determined using enzyme-linked immunosorbent assay (ELISA) kits according to the manufacturer’s protocols (R&D Systems, Minneapolis, MN, USA). The micro-plate provided has been pre-coated with specific antibody, then standards or samples were added to the appropriate microtiter plate wells with biotin-conjugated antibody specific for these factors and avidin conjugated to horseradish peroxidase was added to each microplate well and incubated. Then a substrate solution was added to each well. Only those wells that contain specific factors will exhibit a change in color. The enzyme-substrate reaction was terminated by the addition of a sulphuric acid solution and the color change was measured spectrophotometrically at a wavelength of 450 ± 2 nm. The concentration of the growth factors was then determined by comparing the OD of the samples to the standard curve.

### Injection technique

**Fig. 3:**
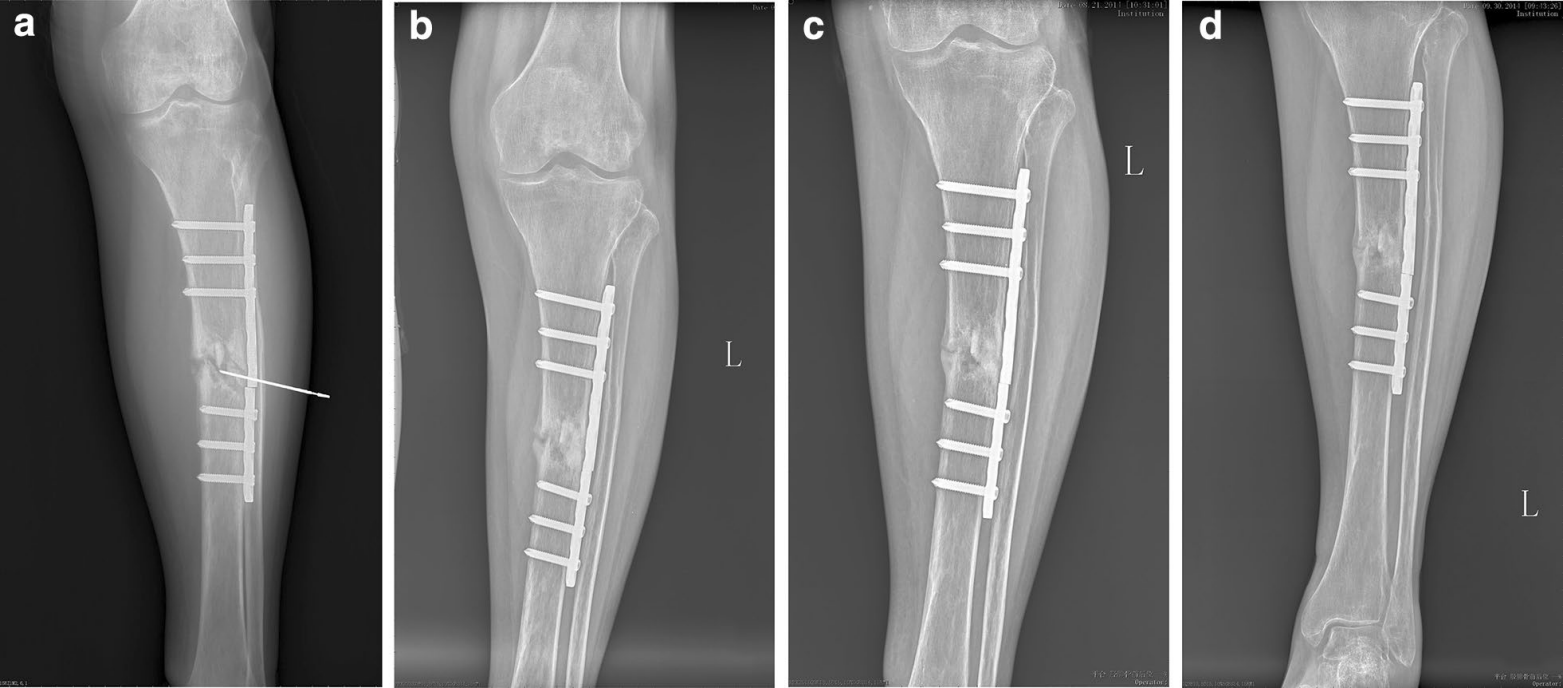
The APL injection and position fixing (**a**), radiographs of tibia fracture at 2 months (**b**), 4 months (**c**) and 6 months (**d**) after injection

The injection was carried out once a week with three injections in one course of treatment. The injection procedure was carried in the operation room under the fluoroscopic guidance. The patient was asked in the supine position with combined spinal–epidural anesthesia. The broken ends of the fracture were confirmed under C-arm and a small needle knife was inserted to the fracture site. After the dissection and brisement of fibrous scar tissue and sclerotic tissue, a disposable needle was inserted perpendicularly into periosteum at the gap of delayed union under fluoroscopic guidance, and then 5 mL of the buffered APL was injected into the area of abnormality (Fig. 3a).

### Post-injection protocol

Prophylactic antibiotics were routinely used at the first 48 h after injection. Weight bearing was prohibited within 24 h after injection. Painless functional training was initiated the next day. Partial bearing was allowed after 3 weeks. After 8 weeks, full bearing was allowed. The use of nonsteroidal anti-inflammatory medication was prohibited during the first 4 weeks after injection. Radiologic evaluation was carried out every month after operation. Routine anteroposterior and lateral tibial X-rays were performed at every subsequent visit to estimate the union healing situation.

### Statistical analysis

The data was presented as the mean ± SD. A Student’s t test was used to determine the significance between the groups. P values of <0.05 were considered significant. All statistical analyses were performed with the SPSS (Statistical Package for the Social Sciences) 13.0 software.

## Results

The factor level detection results (Table 1) showed that the concentration of PDGF-BB, TGF-β1, IGF-1 and EGF in APL were significantly higher than those in the whole blood (P < 0.05), and most of the detected factors increased at least three times.

**Table 1:** The detection of four cytokines concentration in whole blood and APL

Type	Cytokines			
	PDGF-BB (pg/mL)	TGF-β1 (pg/mL)	IGF-1 (pg/mL)	EGF (pg/mL)
Whole blood	138.56 ± 50.64	3.26 ± 0.98	16.45 ± 9.57	128.36 ± 30.36
APL	589.57 ± 96.57	6.59 ± 1.26	118.65 ± 28.56	289.54 ± 68.26
*t*	7.16	3.61	5.88	3.74
*P*	0.002	0.02	0.004	0.02

Two, four and six months after platelet lysates treatment, X-rays showed gradual and abundant callus formation in the tibia fracture site (Fig. 3b–d). More remarkable clinical evidence was shown as there was obvious osseous collection in the tibia fracture site in 6 months after injection (Fig. 3d). The patient reported minimal pain and bony union was almost achieved in 8 months after injection.

**Fig. 4:**
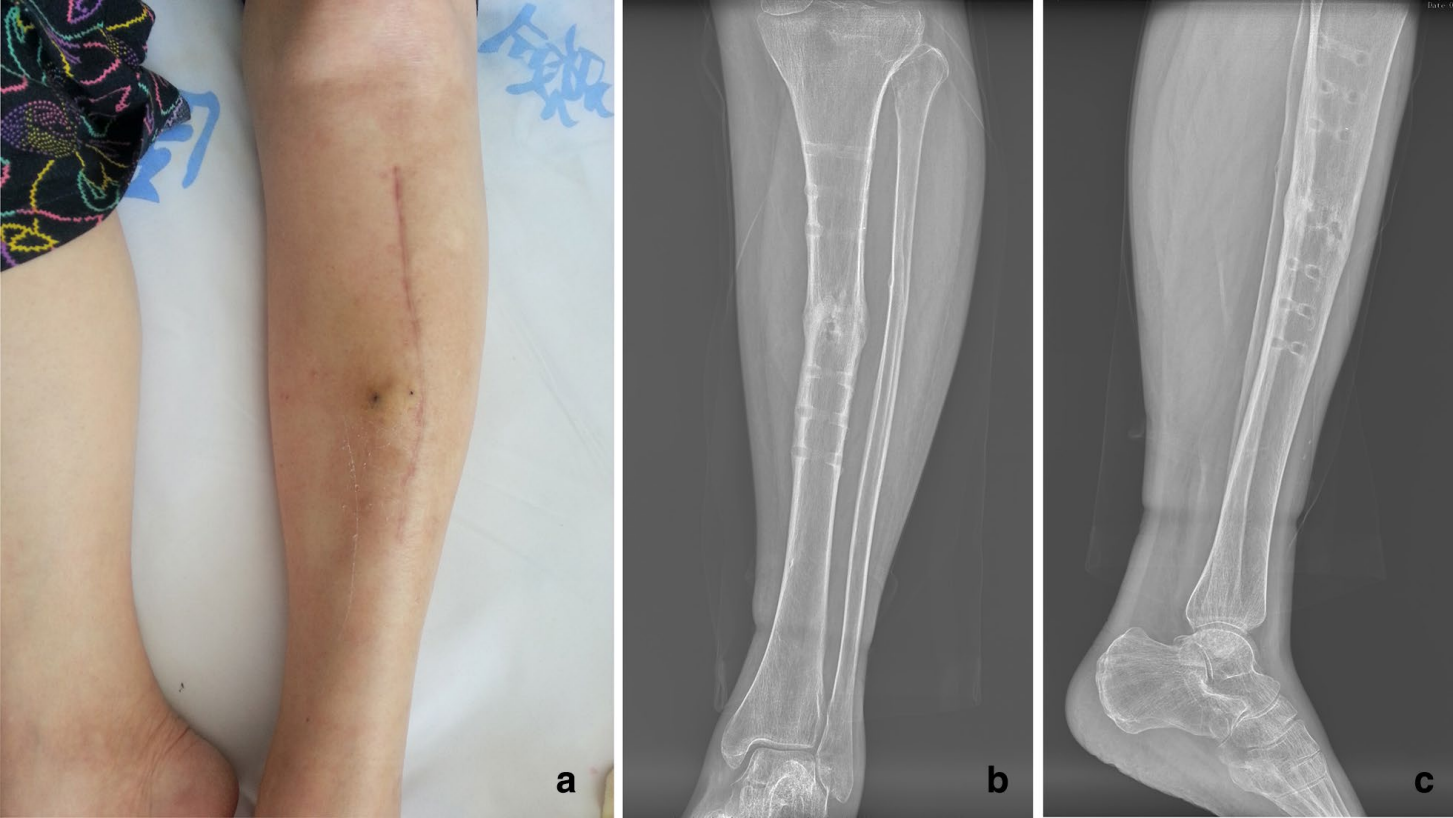
The appearance of the leg after APL injection (**a**), the anteroposterior film (**b**) and lateral film (**c**) after remove the plate at 12 months after injection

The patient didn’t feel any post-injection discomfort, no complications such as infection, refracture etc. were observed. Bony union was achieved after demolishing the nickelclad at in 12 months after injection (Fig. 4c, d). The patient has returned to her normal activity without pain.

## Discussion

Approximately 5–7% of fractures sustained in China were associated with delayed healing or nonunion. As tibia and fibula have less soft tissue cover and are vulnerable, and meanwhile the fracture could cause severe damage to blood supply, they are the most possible sites to cause nonunion or delayed union. The continuous movement of the fracture broken ends could lead to nonunion with persistent pain and seriously reduce the patients’ life quality.

Bone healing is a complex process and the factors interfering bone healing mainly include large fracture gap, inadequate blood supply, unstable internal/external fixation, imperfect postoperative rehabilitation and so on. A wide variety of management options exist for the treatment of tibia nonunion. Radical debridement as well as fixation is the most common method. Other strategies such as internal/external fixation, biological treatment, and physical therapy have been devised to deal with bone fracture but these methods may cause complications such as infection, malunion, nonunion, delayed union and hypoesthesia. It is demonstrated that the intervention of a combined approach is likely to produce the best clinical result.

Autologous platelet products as a source of healing factors have been shown to promote bone tissue repair in orthopedic surgery (Civinini et al. 2011; Mariconda et al. 2008). Memeo et al. (2014) reported a case series of seven young patients with forearm post traumatic nonunion treated by intramedullary nailing and platelet-rich plasma. All patients were identified as being recovered through X-ray assessment and the average recovery time was 23 weeks. The results demonstrated that PRP combined with intramedullary nailing therapy was efficient without the necessity to take autologous bone graft. Another study reported that PRP and mesenchymal stem cells mixed with demineralized bone matrix was also a safe and efficient alternative to autologous bone graft, which achieved great clinical effect in treating distal tibial fractures delayed union (Liebergall et al. 2013). Galasso et al. (2008) conducted a clinical study with 22 non-union patients, and used the combination of expandable intramedullary nailing and PRP to treat them. The results showed that 91% of patients attained bone union at the final follow-up and the average time to union was 21.5 weeks, and no complication was found in this study.

Bielecki et al. (2008) reported a 32 participants’ clinical research to estimate the efficacy of percutaneous autologous platelet-leukocyte-rich gel (PLRG) injection for treating delayed union and nonunion. The investigation showed that the PLRG injection method was sufficient to obtain union. In the healed 25 cases, the average time to union was <11 weeks. This method may become a minimally invasive alternative to incision grafting techniques. But not all patients were cured in this study. This may be related to the high level of white blood cells in the PLRG. Several studies suggested that leukocytes could cause pro-inflammatory effects due to the presence of proteases and acid hydrolyses (Lana et al. 2014; Cerza et al. 2012). Thus the use of PRP containing high level of leukocytes may be questioned.

As a kind of product obtained after repeated freeze-thawing of PRP, APL contain a myriad of bioactive growth factors such as VEGF, insulin-like growth factor, transforming growth factor (TGF-β) etc. (Fekete et al. 2012); these histo-promotive substances could influence tissue repairing through activation of proliferation and chemotaxis of osteoblasts and preosteoblasts, endothelial cells, reconstruction of extracellular matrix and regeneration of new bone (Eren et al. 2016). Besides, through centrifugation and enrichment, APL has few leukocytes and inflammatory reaction may be decreased (Yin et al. 2016).

A rat model had been established by Song et al. (2009) to estimate the effect of platelet lysates upon allogeneic bone reconstruction. Radiology, histology, immunology and biomechanics evaluation indicated that platelet lysates was capable of accelerating the regenerative repair of bone defect and promoting the bone regeneration and osetointergretion of allograft bone after transplantation, which was in coincidence with our study. Only a few clinical studies about the use of platelet lysates in bone healing have been investigated so far.

The purpose of this case report was to establish a better way to promote bone regeneration and find a less invasive technique so that a further surgical procedure could be avoided. The case reported herein is a tibia and fibula delayed union in which surgical treatment failed. In particular, due to improper internal fixation or premature weight bearing, the nickelclad broke up. After consideration of all these points, the APL injection therapy was used for the treatment of the postsurgical delayed union patient. To the best of our knowledge, this is the first reported case of tibia delayed union with breakage of the plate resolved with APL injection.

## Conclusion

In this study, percutaneous injection of concentrated platelet lysates were used for the treatment of a delayed union patient with a broken-up nickelclad. This method was proved to be effective in fracture healing, which may offer the surgeon and patient a new therapeutic option for the treatment of nonunion or delayed healing fractures. Further research and clinical series will hopefully clarify the clinical effect of APL combined with stem cells and allograft for bone defect.
